# Gelsemium Sempervirens in Neuralgia

**Published:** 1876-11

**Authors:** 


Gelsemium Sempervirens in Neuralgia.—Dr. Jurasz, of Heidelberg, has used the tincture in five cases of neuralgia. In the first case, five drops, three times a day (facial neuralgia); cured in three days. Second case, of the same kind as the first, four drops three times a day; cured in six days. Third case, supra-orbital neuralgia, ten drops three times a day; cured in four days. Fourth case, neuralgia of the fifth pair on both sides, five drops every day; cured in two days. Fifth case, severe sciatica, eight drops three times a day; almost cured in fifteen days, and complete cure was then obtained by the continuous current and warm baths. The gelsemium failed to relieve a hemicrania of old standing, and was also unsuccessful in two cases of muscular rheumatism.—*Nash. Jour.*

